# Visually driven functional MRI techniques for characterization of optic neuropathy

**DOI:** 10.3389/fnhum.2022.943603

**Published:** 2022-09-09

**Authors:** Sujeevini Sujanthan, Amir Shmuel, Janine Dale Mendola

**Affiliations:** ^1^Department of Ophthalmology and Visual Sciences, McGill University, Montreal, QC, Canada; ^2^Departments of Neurology, Neurosurgery, Physiology and Biomedical Engineering, Montreal Neurological Institute, McGill University, Montreal, QC, Canada

**Keywords:** glaucoma, optic neuritis, traumatic optic neuropathy, visually-driven fMRI, LGN, visual cortex, MT, LOC

## Abstract

Optic neuropathies are conditions that cause disease to the optic nerve, and can result in loss of visual acuity and/or visual field defects. An improved understanding of how these conditions affect the entire visual system is warranted, to better predict and/or restore the visual loss. In this article, we review visually-driven functional magnetic resonance imaging (fMRI) studies of optic neuropathies, including glaucoma and optic neuritis (ON); we also discuss traumatic optic neuropathy (TON). Optic neuropathy-related vision loss results in fMRI deficit within the visual cortex, and is often strongly correlated with clinical severity measures. Using predominantly flickering checkerboard stimuli, glaucoma studies indicated retinotopic-specific cortical alteration with more prominent deficits in advanced than in early glaucoma. Some glaucoma studies indicate a reorganized visual cortex. ON studies have indicated that the impacted cortical areas are briefly hyperactive. For ON, brain deficits are greater in the acute stages of the disease, followed by (near) normalization of responses of the LGN, visual cortex, and the dorsal visual stream, but not the ventral extrastriate cortex. Visually-driven fMRI is sensitive, at least in ON, in discriminating patients from controls, as well as the affected eye from the fellow eye within patients. The use of a greater variety of stimuli beyond checkerboards (e.g., visual motion and object recognition) in recent ON studies is encouraging, and needs to continue to disentangle the results in terms of change over time. Finally, visually-driven fMRI has not yet been applied in TON, although preliminary efforts suggest it may be feasible. Future fMRI studies of optic neuropathies should consider using more complex visual stimuli, and inter-regional analysis methods including functional connectivity. We suggest that a more systematic longitudinal comparison of optic neuropathies with advanced fMRI would provide improved diagnostic and prognostic information.

## Introduction

The term *optic neuropathy* refers broadly to injury to the optic nerve. It is a serious condition that may result in loss of visual acuity and/or deficits to the visual field (partial or even full blindness). This type of injury can develop acutely or chronically. Acute optic neuropathies such as optic neuritis (ON) and traumatic optic neuropathy (TON) have a rapid onset and are typically caused by inflammation and trauma, respectively, whereas chronic optic neuropathies such as glaucoma are characterized by slow progression (Behbehani, 2007). The aim of this review is to determine how visually-driven functional MRI (fMRI) can be used to assess optic neuropathy-related effects on the entire visual system and potentially provide differential diagnostic or prognostic value in clinical settings. In a separate review, we review resting-state fMRI studies for the same patient groups. By comparing and contrasting glaucoma and ON we can begin to identify common or differential abnormalities induced by chronic vs. acute optic neuropathy. Glaucoma is a prominent example of a disease with progressive effects within the brain as a result of persistent impairment to visual input. In contrast, ON, quickly and severely disrupts normal retinal input but subsequently stabilizes, and recovery of function is common. These well-studied diseases can serve as an important benchmark for comparison and indication of what might be possible for a related optic neuropathy, TON, for which there are no fMRI studies thus far. In addition, ON studies will aid in understanding brain abnormalities specifically due to central visual field loss, which is what is expected—at least initially—in TON patients. We aim in particular to suggest that visually-driven fMRI techniques might contribute to further characterize TON and subsequently aid in the diagnosis, especially in the case of bilateral trauma when the diagnosis is most challenging. We first define some terminology related to the current standard methods of diagnosis and staging of neuropathies. Next, we define the fMRI technique that allows measurement of visual system function. Subsequent sections offer a critical review of published studies that used visually-driven fMRI to assess optic neuropathy-related abnormalities in thalamic and visual cortical areas. Glaucoma, ON and TON are each discussed separately.

### Ophthalmologic assessment tools

**Figure 1:**
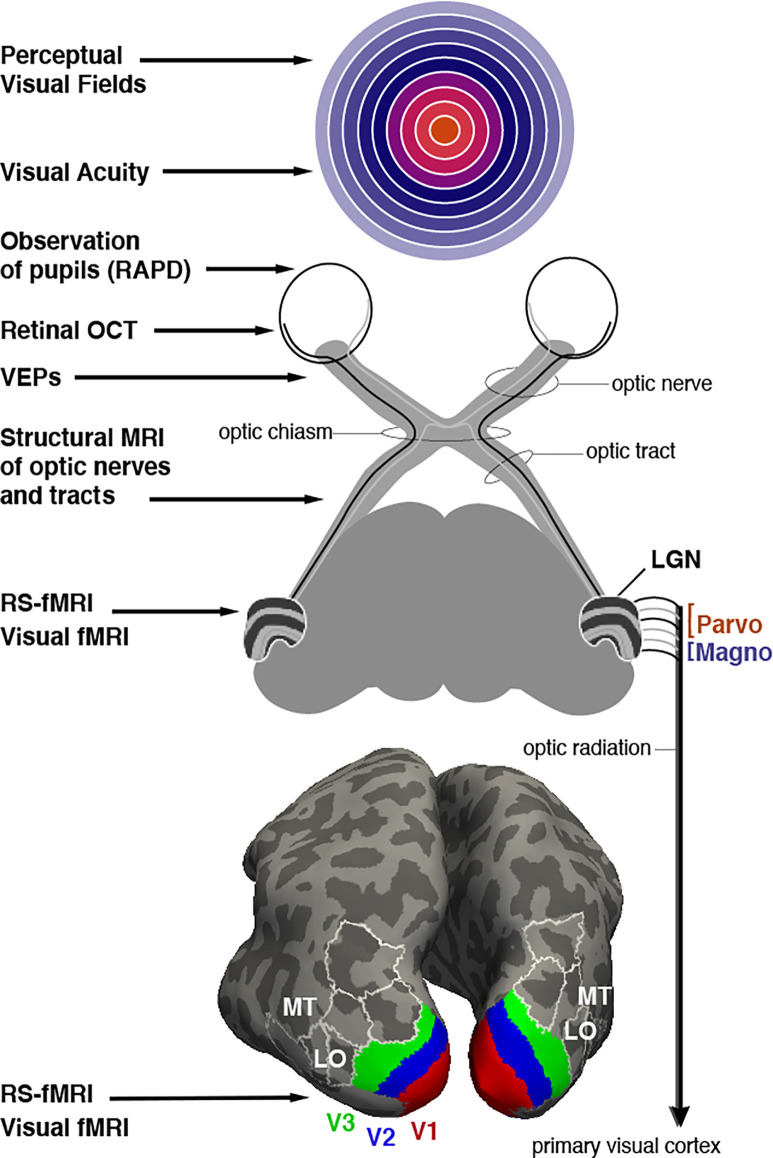
Assessment of optic neuropathy at different levels of the visual system. Potential ophthalmological tools are listed that rely on behavioral testing, clinical observation, microscopy, electrophysiology or MRI imaging (on left). **Top image**: Representation of the visual field with (foveal) central vision colored orange, mid-eccentricities colored with cooler hues, and peripheral locations colored with increasingly light purple. White rings represent eccentricities spanning from 10 to 90 degrees in 10 degree increments. **Middle image**: Schematic description of retinal projections to the thalamic lateral geniculate nucleus (LGN). LGN is shown with layers 1–6 that segregate input from each eye (contra, ipsi, ipsi, contra, ipsi, contra). Layers 1 and 2 form the magnocellular projections to V1 while layers 3–6 comprise the parvocellular input. **Bottom image**: Inflated depiction of cerebral cortex, posterior view. A probabilistic brain atlas (Wang et al., 2015) is used to show the location of several retinotopically organized visual areas. Lower-tier areas V1, V2, and V3 are shown with red, blue, and green, respectively. Some additional higher-tier areas are indicated as well, in particular areas MT and LO (LO1 and LO2) mentioned in the text as well. White outlines, included for context, localize area V3A, V3B, IP0 (dorsally), and V4v, VO1, VO2 (ventrally).

In this section, we briefly catalog measures from common ophthalmological tools that are often considered in fMRI literature as covariates that may relate neuroimaging markers to levels of disease severity. A summary figure lists these tools in relation to applicable levels of the visual system (Figure 1). The primary tools used for determining the extent of visual function loss in optic neuropathies evaluate structural defects in the retina and optic disc, and/or functional impairments in vision. Aspects of retinal anatomy such as the retinal nerve fiber layer (RNFL) thickness, or morphology of the optic nerve head (e.g., rim area and volume, as well as cup-disc ratio) are measured using optical coherence tomography (OCT), confocal scanning laser ophthalmoscopy, or scanning laser polarimetry. These tools currently provide valuable information about the severity of the disease but do not differentiate well between different optic neuropathies. Physical examination is accompanied with visual field testing, typically conducted with automated static perimetry, where sensitivity to light is evaluated using a small white flash on a dim background. Both focal and global visual field loss are often utilized for determining the pattern of visual field loss (i.e., predominantly central or peripheral) and the progression of the disease (Susanna and Vessani, 2009; Yaqub, 2012). Due to the retinotopic nature of these tests, there is a strong potential for direct correlation with fMRI measurement of the retinotopic maps in the visual cortex. These behavioral tests are also clinically important but do have limitations (Wilhelm and Schabet, 2015; Wu and Medeiros, 2018). The testing can be subjective, challenging for patients, and has poor sensitivity (e.g., to detect glaucomatous peripheral field loss). Some specific perimetric tests have been developed that attempt to isolate specific types of retinal ganglion cells, such as magnocellular or short-wavelength cell types which are biased in number in the peripheral or central retinal, respectively (Sharma et al., 2008). Although these could again be attractive as potential covariates for neuroimaging measures, they have not been widely successful clinically in identifying all individuals at risk for developing glaucoma (Havvas et al., 2013). Visual acuity, an eye’s ability to resolve high spatial resolution, is often a critical measure obtained with Snellen or Early Treatment Diabetic Retinopathy Study charts. It can quickly estimate the quality of central vision, which to some extent serves as a measure of disease severity in optic neuropathies (Toosy et al., 2014). Traditionally, results from multiple longitudinal eye examinations are utilized for diagnosis and staging of patients. Furthermore, there are also other functional vision measures that provide valuable information about certain optic neuropathies. For example, visually evoked potentials (VEPs), a measure of the integrity of the retino-geniculate-cortical pathways, is strongly associated with the deficits in motion perception observed in ON patients. The relative afferent pupillary defect (RAPD) is another important and widely used ophthalmological assessment to determine asymmetry in reflexive pupillary response when light is alternately applied to each eye. The objective nature of the test makes it convenient to use, especially in trauma patients with limited responsiveness. However, it is only useful in identifying optic neuropathies that are unilateral or asymmetric in nature (Broadway, 2012). Correlation analysis between functional neuroimaging variables and these key clinical measures can help identify the most clinically relevant fMRI markers of injury for each optic neuropathy.

### Functional MRI techniques

fMRI is a non-invasive method to measure neuronal activity across the brain. Changes in neuronal activity are measured indirectly with the blood oxygenation level-dependent signal (BOLD; Kwong et al., 1992; Ogawa et al., 1992). fMRI is useful to vision science because we can address fundamental questions such as the location of the multiple representations of the retinal “map” in the occipital, posterior parietal, and posterior temporal lobes of the brain (e.g., V1, V2, V3, etc., as shown in Figure 1). It is also possible for subjects to perform a wide array of visual detection or discrimination tasks so that the most actively recruited cortical regions are visualized. Questions such as the degree of specialization of certain cortical areas can be addressed. Since fMRI has no known risk to subjects, they can be scanned repeatedly to monitor change over time. This can be exploited to study perceptual learning in healthy controls, for example. fMRI offers a wide field of view, and can easily cover the entire brain, although a trade-off exists between spatial resolution and field of view. In other words, to achieve the smallest voxels possible, a smaller volume of the brain is imaged. In this sense, the field is still limited by a finite spatial and temporal resolution. Using very advanced MRI scanners at ultra-high field strengths (like 7 Tesla) helps achieve even better spatial resolution (Chaimow et al., 2018). The temporal resolution of fMRI is inherently limited to approximately 1–2 seconds; it does not compete with electrophysiological techniques such as VEPs measured in milliseconds. Those techniques are thus complementary.

There are other practical limitations to fMRI that derive from experimental design and analysis. For example, should the visual stimuli remain identical, or should the levels of visual performance remain identical across a range of subjects? In addition, in the context of clinical studies, it is very desirable to be able to make reliable fMRI measures in individual subjects so as to discriminate a patient from healthy controls. This is already possible, but sensitivity may be limited compared to comparisons at group level. All the studies reviewed here made comparisons at the group level. Emerging advances in data analysis may prove beneficial for improving single-subject analyses. For example, probabilistic atlases (from normal healthy subjects) can be applied to an individual subject’s data to estimate the retinotopic boundaries of many visual areas, even if retinotopic mapping has not been performed for that particular subject. In the future, we expect that even more powerful analysis methods from the machine learning field can be employed to maximize sensitivity for classification (e.g., of diagnosis) of visual impairment and prediction (e.g., of prognosis) of recovery in an individual patient.

It is pertinent to this clinically-oriented review that some features of fMRI have been a persistent limitation when translation to clinical ophthalmological settings is considered. Importantly, stimulation of the peripheral field can be challenging inside the bore of MRI machines. In most studies the stimuli remain within the central 20 degrees. Specialized equipment is needed to reach the far periphery. In order to circumvent this limitation, one probabilistic atlas has cleverly estimated the locations of V1–V3 out to 90 degrees (Benson and Winawer, 2018). Moreover, specialized hardware is also required to allow separate images to be shown to each eye during fMRI sessions (i.e., dichoptic stimulation). Currently, there are multiple products on the market for dichoptic presentation of visual stimuli and thus allow for fMRI responses of each eye to be directly correlated with eye specific clinical measures. fMRI experiments typically require 1–2 hours with little to no movement, which may be considered demanding for patients. Visually-driven fMRI requires subjects to remain alert with eyes open, and usually subjects should avoid eye movements by fixating their gaze on a central mark for several minutes at a time. Finally, there is a serious commitment of time and expertise required to analyze and interpret the results.

**Figure 2:**
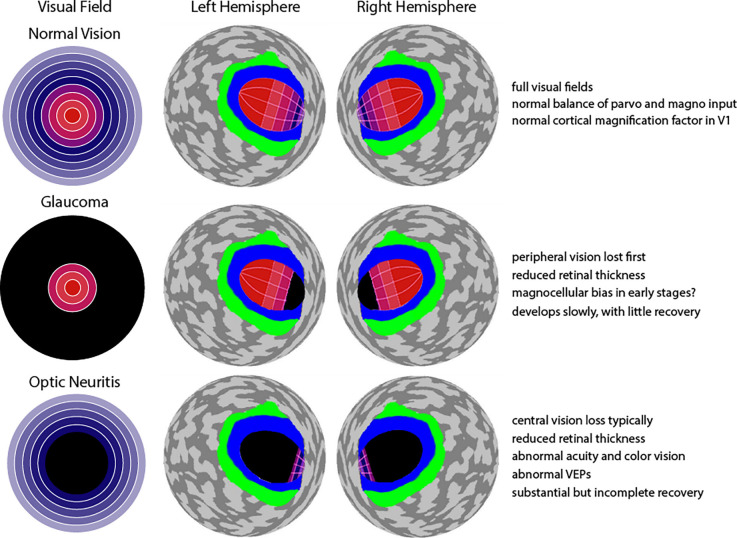
Effect of glaucoma and optic neuritis on visual field and visual cortex. **Top row**: A normal visual field diagram with orange and purple signifying central and peripheral vision, similar to Figure 1 is shown. To the right is an abstracted (spherical) representation of both the left and right hemispheres of cerebral cortex, posterior view (Benson and Winawer, 2018). Area V1 is labeled with the same orange-to-purple color gradient used for the visual field. It can be seen that the central vision occupies a disproportionally large region of V1, with very little territory devoted to the periphery. A similar representation also exists in areas V2 and V3, but this is not shown. Instead, area V2 is simply shown in blue, and area V3 in green. **Middle row**: Loss of peripheral vison in glaucoma leads to deafferentation of a relatively small portion of anterior V1 (shown with black) due to the cortical magnification factor. **Bottom row**: Loss of central vison in optic neuritis leads to deafferentation of a quite large proportion of V1 (shown with black) due to the exponential expansion of central field input. Text in far right summarizes features for normal vision, glaucoma, and optic neuritis. Note: depiction of cortical magnification factor in V1 remade from a source at https://mosaicofminds.medium.com/maps-in-the-brain-f236998d544f.

Retinotopic mapping, the process of determining the visual receptive field properties of neuronal populations in the visual pathway (i.e., how the brain maps our visual field), is possible with BOLD fMRI, and is a useful technique to investigate the differences in visual system organization of healthy individuals and patients. Retinotopic mapping typically uses expanding ring stimuli to measure eccentricity and rotating wedge stimuli to measure polar angle (Sereno et al., 1995). The brain contains multiple, topographically organized, maps of visual space in the early visual areas, e.g., V1, V2, and V3, where neighboring visual space is represented in neighboring regions in visual cortex. Central foveal vision in particular is represented in multiple regions including the occipital pole, as well as the lateral occipital cortex (LOC; Wandell et al., 2007). Another important feature is cortical magnification: a large portion of the visual cortex is dedicated to processing information from the central visual field, and as the distance from the fovea increases, cortical volume decreases exponentially (Wandell et al., 2007). Importantly, any changes to this normal pattern of cortical activation may suggest an impairment, compensatory effect (i.e., higher activity than normal), or even cortical reorganization. Moreover, compared to damage to peripheral vision, damage to central vision is expected to result in abnormal representation across a much larger expanse of visual cortical areas (Figure 2). This is a dramatic effect that would apply to many different visual areas, but this is depicted only for V1 in Figure 2.

## Methods

We searched PubMed using keywords specific to these optic neuropathies and selected peer-reviewed articles in English with relevant titles and abstracts for further review. Search terms included glaucoma, primary open-angle glaucoma, optic neuritis, optic neuropathy, optic damage, traumatic optic neuropathy or open globe injury, and keywords related to fMRI. After the initial selection, the full text was assessed for eligibility. We included studies containing patient groups affected by POAG, acute ON or clinically isolated syndrome of ON, as well as vision loss due to trauma to the optic nerve (i.e., open globe injury or TON). We specifically focused on typical idiopathic ON. Other related inflammatory diseases of the visual system (e.g., neuromyelitis optica spectrum disorder, MOG-antibody ON, lupus, or GPA) were excluded as there are no visually-driven fMRI studies for such patients. Moreover, ON studies consisting primarily of MS patients were excluded. More details about the clinical profiles of ON patients utilized in the studies we reviewed can be found in Supplementary Table 1. Studies that focused on any other optic neuropathy, hereditary vision damage, animal research, molecular work, and clinical studies (i.e., case reports, randomized controlled trials and clinical trials) were also omitted. In addition, in this review we only included studies using visually-driven fMRI to investigate responses in cortical and subcortical visual areas of the brain. We chose to focus exclusively on the visual system in order to better isolate those findings and examine the untapped potential for improved sensitivity to the direct effects of deafferentation in this system.

## Glaucoma

Glaucoma is a leading cause of permanent blindness worldwide and has blinded about 3.2 million people as of 2020 (Broadway and Cate, 2015). This chronic and generally bilateral neurodegenerative optic disease leads to progressive visual field loss, typically with the peripheral visual field affected in the early stages, followed by central visual field loss. Partial regions of blindness develop gradually such as nasal step scotoma, inferior or superior arcuate scotoma, paracentral scotoma or generalized depression (Weinreb and Khaw, 2004; Jonas et al., 2017). This is a result of damaged retinal ganglion cells and degeneration of the optic nerve, producing characteristic changes to the optic nerve head known as “cupping” (Cohen and Pasquale, 2014). It may or may not be associated with high intraocular eye pressure, but this is currently the only modifiable risk factor for managing glaucomatous symptoms, and glaucoma may continue to progress following treatment. One of the most common types of glaucoma and the one that will be discussed in this paper is POAG.

Progressively, glaucoma results in optic nerve “rim” loss, increased cup to disc ratio, disc hemorrhage, retinal nerve fiber damage, and enlarged visual field defects. However, the biological mechanisms and sequela underlying these clinical presentations are poorly understood. Moreover, although these features are important in the diagnosis and follow-up of glaucoma, they cannot be used to define glaucoma with high sensitivity and specificity (Cohen and Pasquale, 2014). POAG is asymptomatic (i.e., normal visual field) until a substantial number (i.e., 25%–35%) of retinal ganglion cells have been permanently lost and visual deficits start to form within the central visual field (10°–20° degrees from fixation; Qing et al., 2010). Therefore, individuals are recommended to have their eyes examined periodically to detect structural changes to the optic disc, one of the first signs of glaucoma detectable in clinical eye examinations.

With the increasingly wide availability of functional MRI techniques, recent studies have started to examine the structure and function of the lateral geniculate nucleus (LGN), and visual cortex in glaucoma patients to observe any other signs of alterations that may provide new knowledge about the disease and serve as promising avenues to detect and halt vision loss. Indeed, many studies have documented injury to the optic nerve, and tract, as well as abnormalities in visual and even non-visual subcortical and cortical regions and networks (Lawlor et al., 2018). Degeneration along the visual pathway is believed to be triggered by the loss of retinal ganglion cells in a process called transsynaptic degeneration, where injured neurons subsequently impair their projection targets, depriving them of input connections. Investigating the changes in brain activity in the visual areas of glaucoma patients may help further understand the pathophysiology of the disease, and potentially aid in the development of interventions.

### Visually driven functional brain measures

**Figure 3:**
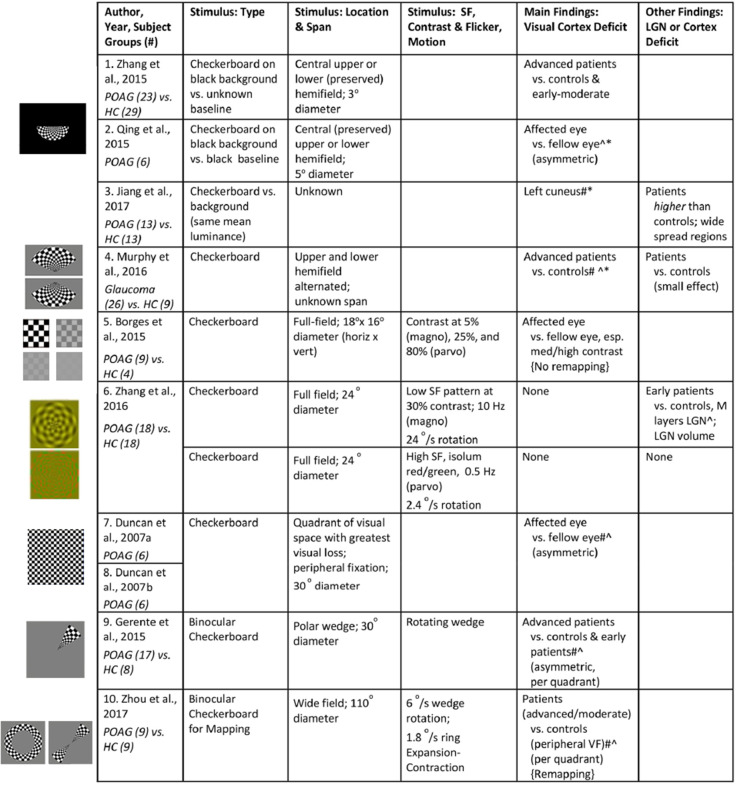
Summary of visual stimuli utilized and findings in visually-driven fMRI studies of glaucoma. The studies are listed in increasing order of the size of the checkerboard stimulus utilized. Left: Images of visual stimuli, similar to the ones utilized in each of the studies, have been provided. Note: Images in row 1, 2, 4, 9, and 10 are created with a figure licensed under a Creative Commons Attribution 4.0 International (https://creativecommons.org/licenses/by/4.0/, https://creativecommons.org/licenses/by/4.0/legalcode). It is attributed to Biological Psychiatry (https://www.sciencedirect.com/science/article/pii/S0006322307012413?via%3Dihub#fig1). Cropped in various shapes from original. Images in row 6 is obtained from Zhang et al. (2016) © 2015 Wiley Periodicals, Inc. ^∧^Deficit indicated by correlation with visual sensitivity measures (i.e., BOLD activity declines with increasing severity measure). *Compensation indicated by correlation with visual sensitivity measures (i.e., BOLD activity increases with increasing severity measure). ^#^Deficit indicated by correlation with retinal thickness (i.e., BOLD activity declines with increasing severity measure). ^#*^Compensation indicated by correlation with retinal thickness (i.e., BOLD activity increases with increasing severity measure). SF, spatial frequency; VF, visual field; POAG, primary open-angle glaucoma; HC, healthy controls.

We identified 10 cross-sectional visually-driven fMRI studies investigating the effect of glaucoma on the visual system. The stimulus parameters utilized and results reported in these studies are summarized in Figure 3. Unless otherwise indicated in column *Stimulus: Type* in Figure 3, the studies monocularly presented a standard checkerboard stimulus which consists of central fixation, black and white checkers with 100% contrast, and contrast reversing at 8 Hz to the affected eye. The stimulus is generally presented in a block design where it is alternated with a blank background with the same luminance as the mean luminance in the stimulus period. The fellow eye is generally patched in all studies except in Duncan et al. (2007a, b) where it was voluntarily closed, and fellow eye status is unknown for in Jiang et al. (2017). All glaucoma studies scanned patients at 3 Tesla and the resolution of the functional scans across the studies ranged from 2 to 3.4 mm^3^. Moreover, visual areas seem to be delineated using retinotopy (Duncan et al., 2007a, b; Borges et al., 2015; Zhang et al., 2016; Zhou et al., 2017) or an automated anatomical labeling system (Gerente et al., 2015; Jiang et al., 2017), and data were analyzed in Talairach (Borges et al., 2015) or Montreal Neurological Institute (Qing et al., 2010; Zhang et al., 2015, 2016; Murphy et al., 2016; Jiang et al., 2017) reference space.

The current literature indicates that visually driven fMRI has the ability to distinguish glaucoma patients from healthy subjects. For example, Borges et al. (2015) observed a reduction in activity in the bilateral occipital lobe in response to stimulation of the glaucomatous eye in POAG patients compared to healthy controls. In addition, it is interesting to note that the functional loss was only evident for the medium and high contrast stimuli and not for low contrast stimuli (Borges et al., 2015). Furthermore, eccentricity-specific reduction in the activity of the bilateral occipital lobe has been reported. Zhou et al. (2017) utilized task-based fMRI and confirmed that the stimulation of the peripheral visual field resulted in a lower BOLD percent change compared to control subjects. However, the results have been mixed regarding the degree of abnormality in the *central* visual field. Zhou et al. (2017) found no difference in central-field visual regions between a cohort of POAG patients with various levels of disease severity and controls. However, on the contrary, presenting a central checkerboard stimulus to a relatively preserved central visual field did result in a reduction of BOLD percent change in asymmetric glaucoma patients (Qing et al., 2010), and did show compromised cerebral blood flow in central field visual cortex of advanced POAG patients (Zhang et al., 2015). Although the reason for these discrepant results is uncertain, one likely source would be variability in the staging and severity of disease in the respective patient groups studied.

There is more direct evidence that the extent of change in visual pathways and cortical activation is dependent on glaucoma severity across the spectrum from early to advanced disease. In early stages, visual field score seems to remain relatively normal despite reductions in RNFL thickness (Bowd et al., 2001), and indeed BOLD percent response in the visual cortex of the early to moderate POAG participants was not significantly abnormal (Gerente et al., 2015; Zhang et al., 2015; Zhou et al., 2017). Likewise, Zhang et al. (2016) reported a similar phenomenon but also suggested that early POAG patients may have a biased loss of *magnocellular* input compared to controls, which is consistent with the early loss of peripheral vision where magnocellular input dominates. In particular, early-stage glaucoma patients showed a reduced fMRI response in LGN to transient achromatic stimuli compared to sustained chromatic stimuli, suggesting loss of the magnocellular channel. While these findings seem promising, it is important to replicate these results using even newer methods that provide the high spatial resolution required to confidently isolate BOLD signal in individual layers of the LGN. When severe stages of glaucoma are studied, activation in the calcarine and occipital cortex is low compared to healthy controls particularly in the anterior calcarine sulcus where the representation of peripheral field is expected (Zhang et al., 2015; Murphy et al., 2016). Murphy et al. (2016) suggest that this retinotopic pattern of functional loss is more strongly observed in the primary visual cortex in comparison to higher visual areas, but the data is still lacking to show this clearly. It remains to be determined if extrastriate retinotopic visual cortices could ever be more sensitive to subtle alterations in visual function than any of the clinical parameters currently used for diagnosis with appropriately tailored stimuli and optimized fMRI protocols.

We conclude this section by mentioning that, despite the consistent reduction in functional brain activity as a result of POAG, some authors have argued that the visual cortex may actively compensate for the loss of visual input via specific reorganization. In particular, remapping may occur in early visual cortices to compensate for the reduced vision in the peripheral visual field. In other words, the foveal or parafoveal representations may be strengthened, possibly by recruiting deafferented cortical territory formerly representing more peripheral eccentricities. Indeed, parafoveal regions in the visual cortex were found to be enlarged in mild to moderate POAG patients compared to controls in V1 and V2, but not V3 (Zhou et al., 2017). However, Borges et al. (2015) reported that they noticed a lack of remapping in affected regions of V1 and V2 in glaucoma in patients with *unilateral* POAG. This might suggest that reorganization does not occur until cortical circuits (which are primarily binocular) are consistently deafferented from both eyes. Finally, we note that Jiang et al. (2017) used a checkerboard stimulus and reported compensatory-like activity (i.e., higher BOLD signal) in various structures involved in the limbic network, default mode network, the dorsal visual pathway, and frontal gyri, of patients compared to controls. This seems to suggest that the effects of glaucoma are widespread, however this needs to be further explored with stimuli that specifically and maximally drive the specialized visual and extrastriate regions.

### Correlation with specific ophthalmological measures

Consistent with the strong relationship between fMRI results and overall measures of disease severity, many, but not all, studies report the expected significant correlations between more specific clinical measures and BOLD response in certain cortical areas. BOLD response in visual cortical areas of POAG patients showed a strong, positive correlation with RNFL thickness measured with OCT (Duncan et al., 2007a; Gerente et al., 2015) in the corresponding quadrant (Zhou et al., 2017), but not GDx nerve fiber layer analyzer, and mean disc height contour measured with Heidelberg retina tomograph II (Duncan et al., 2007a). Specifically, deterioration of peripapillary RNFL and macular ganglion cell-inner plexiform layer was shown to be strongly associated with lower activity in the primary visual cortex (Murphy et al., 2016) and less strongly associated with the BOLD signal from secondary and tertiary visual cortices (Murphy et al., 2016). Surprisingly, in one study of early glaucomatous stages, BOLD signals from the left cuneus were negatively correlated with relative RNFL (Jiang et al., 2017), suggesting compensatory activity. Also, in one exceptional study, the difference in BOLD response of the glaucomatous and healthy eye as a result of stimulating the *preserved* central visual field of asymmetric POAG patients did *not* correlate to the corresponding RNFL thickness values (Qing et al., 2010). Although the authors speculate that functional changes in the visual cortex may appear before clinical retinal signs, this remains unproven.

Unlike measures of the retina and optic nerve, behavioral perimetry scores from visual detection tests can estimate the output of the entire visual system and may be an even better measure of function. Indeed, the visual deficits determined by sensitivity differences between a glaucomatous and healthy eye per visual field location (Duncan et al., 2007a), or per quadrant (Zhou et al., 2017) have been shown to correlate with reduced BOLD amplitudes in the corresponding calcarine and occipital cortex areas (Gerente et al., 2015). However, in contrast, Borges et al. (2015) reported no correlation between BOLD activation in the lesion projection zones in V1 and V2 of unilateral POAG patients and the corresponding visual field mean deviation scores. Even more surprising, Qing et al. (2010) report an inverse correlation between BOLD response and visual field scores, suggesting compensation. The difference in the results reported might be attributed to the various types of POAG included in the studies mentioned above including bilateral patients with varying degrees of asymmetry and the rare unilateral cases. It may also be relevant that the studies mentioned above employ slightly different methods such as performing visual field testing monocularly (Borges et al., 2015) vs. estimating binocular visual field using monocular tests (Qing et al., 2010; Gerente et al., 2015; Zhou et al., 2017), or stimulating the peripheral (Duncan et al., 2007a; Gerente et al., 2015; Zhou et al., 2017) vs. the *preserved* central visual field (Qing et al., 2010 and Borges et al., 2015). More work is required to disentangle all these factors.

### Visual stimuli

Eight of the 10 reviewed studies focused mainly on the visual cortex and reported significant changes in BOLD activity within V1. This can be attributed to the type of visual stimuli chosen and the subgroup of POAG patients in these studies. BOLD activity within visual cortices was not significantly altered in studies (*n* = 2) that solely used early POAG patients. For all studies, the visual stimuli were predominantly black and white checkerboard stimuli with 100% contrast and contrast reversing at 8 Hz (see Figure 3). These stimulus parameters are known to maximally drive V1 (Fox and Raichle, 1984; Singh et al., 2000). Amongst the studies, the stimulus varied slightly in terms of size, the number of quadrants covered, and whether it was presented to the most preserved or the most affected area (see Figure 3). Nevertheless, BOLD activity in V1 or other occipital ROIs was consistently lower for POAG patients with at least mild-to-moderate severity.

Two of the 10 studies also used more complex checkerboard stimuli, i.e., concentric eccentricity rings or rotating polar wedges (see Figure 3). These were particularly useful in making specific conclusions about a potential reorganization of the visual cortex in different subgroups of POAG patients. Distinctions between early and severe POAG subgroups were successfully made with retinotopic mapping stimuli that spanned 30 degrees in diameter (Gerente et al., 2015). Zhou et al. (2017) studied this more systematically with retinotopic stimuli that spanned up to 110 degrees. Consistent with the previous study, differences between glaucoma patients and controls were seen when a visual field of around 20 degrees eccentricity was stimulated. Nevertheless, it is important to note that the sensitivity to differentiate (at least mild to moderate) patients from controls increases with stimuli that span greater than 24 degrees eccentricity.

## Optic Neuritis

ON is an acute demyelinating optic neuropathy resulting in a temporary vision loss due to inflammation of the optic nerve. The annual incidence of ON in the world is estimated to be 0.94–2.18 per 100,000 (Toosy et al., 2014). The most common form of ON is associated with multiple sclerosis and therefore is often linked to it. However, ON can present in isolation (Toosy et al., 2014). ON usually occurs in young adults and is characterized by mild to severe unilateral visual loss which develops rapidly and is accompanied by pain during eye movements, followed by spontaneous improvement. Initially, the affected eye in ON has diffuse abnormalities in the central visual field leading to reduced visual acuity; an early clinical sign of ON (Keltner et al., 1999; Toosy et al., 2014). However, patterns of visual field loss alone are of limited value in the differential diagnosis of ON and other optic neuropathies (Pau et al., 2011). The Optic Neuritis Treatment Trial also revealed that the fellow eye typically shows minimal to moderate deficits in the majority of ON patients (Keltner et al., 1993). Finally, other aspects of vision that are usually compromised include color vision; both blue-yellow and red-green defects.

The diagnosis of (unilateral) ON often involves assessing the relative afferent pupillary defect (RAPD) to determine asymmetry in an otherwise symmetric reflexive pupillary response. However, this test fails in cases of bilateral and symmetric visual loss (Broadway, 2012), or if there was previous episode of ON. RAPD assesses the function of one eye in comparison to the other eye. Therefore, the test requires patients to have one eye unaffected by optic neuropathies. In the case both eyes are or have been, affected, the test loses sensitivity. Diagnosis is also assisted by imaging the optic nerve with contrast-enhanced MRI to detect inflammation-mediated breakdown of the blood-nerve barrier (Toosy et al., 2014), and OCT retinal measurements of the thickness of the peripapillary nerve fiber layer (Wilhelm and Schabet, 2015). At disease onset, the optic nerve may appear normal, or RNFL thickness may be higher than normal due to the presence of edema, but a reduction in thickness has been identified as ON progresses (Toosy et al., 2014).

Since visual acuity and visual field are reported to eventually recover (almost) completely even after significant structural loss, it is difficult to gauge the permanent visual consequences of inflammation and predict the extent of subsequent recovery with these diagnostic techniques. Indeed, deficits in contrast sensitivity, color vision, and stereopsis, as well as delayed visual evoked potentials (VEP) may continue after recovery, and are thought to persistently deliver an abnormal visual input to the visual cortex (Beck et al., 1992). There is some evidence that shows, regardless of the degree of optic nerve degeneration and/or demyelination after ON, inter-eye VEP amplitude differences start to decrease 3–6 months from disease onset (Brusa et al., 2001). This suggests neural plasticity of posterior visual pathways contributes to the recovery of visual function in ON patients (Chan, 2012). Therefore, analyzing the impact of ON in post-optic nerve structures longitudinally using visually-driven fMRI could provide a better understanding of the consequences of ON at each stage of the visual pathway and may help us explain the observed deficits. It has been noted that functional vision measures (i.e., visual acuity, contrast sensitivity, etc.) at 1 month, not within the first week of ON onset, are good indicators of visual performance at 6 months (Kupersmith et al., 2007). As such, longitudinal fMRI findings from post-optic nerve structures can be of value in developing more accurate predictions about the progression of ON. ON patients are considered to be in the acute phase within 1 month of disease onset, recovery phase at around 3–4 months, and recovered to normal or near-normal at 1-year from disease onset. Patient demographics and clinical characteristics included in the studies reviewed in this article are reported in the supplemental information (see Supplementary Table 1).

### Visually driven functional brain measures

**Figure 4:**
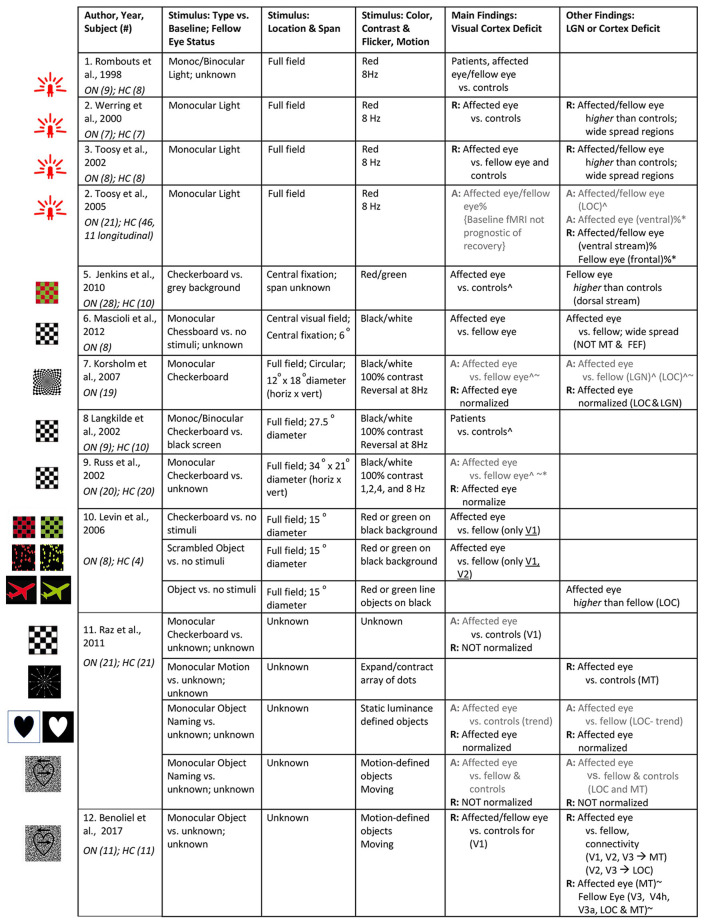
Summary of visual stimuli utilized and findings in visually-driven fMRI studies of ON. The studies are listed based on the type of stimulus utilized. Left: Images of visual stimuli, similar to the ones utilized in each of the studies, have been provided. Note: Image in row 7 is licensed under a Creative Commons Attribution 4.0 International (https://creativecommons.org/licenses/by/4.0/, https://creativecommons.org/licenses/by/4.0/legalcode). It is attributed to Biological Psychiatry (https://www.sciencedirect.com/science/article/pii/S0006322307012413?via%3Dihub#fig1). A: Findings from ON patients in acute stage of disease (i.e., <1 month from disease onset); R: Findings from ON patients in recovery or recovered stage of disease (i.e., >3 months from disease onset); otherwise, results are obtained from mixed group of ON patients or time since onset unknown. ^∧^Deficit indicated by correlation with visual sensitivity measures (i.e., BOLD activity declines with increasing severity measure). ^%^Deficit indicated by correlation with optic nerve structural integrity (i.e., BOLD activity declines with increasing severity measure). ^%*^Compensation indicated by correlation with optic nerve structural integrity (i.e., BOLD activity increases with increasing severity measure). ^~^Deficit indicated by correlation with optic nerve conduction measures (shorter VEP latency/ higher amplitude) (i.e., BOLD activity declines with increasing severity measure). ^~*^Compensation indicated by correlation with optic nerve conduction measures (shorter VEP latency/ higher amplitude; i.e., BOLD activity increases with increasing severity measure). Note: studies mentioned in rows 5–9 did not provide stimulus figures. We have added reference figures based on stimulus description. ON, optic neuritis; HC, healthy controls.

We identified 12 visually-driven fMRI studies investigating the effect of ON on the visual system. Of these, there are cross-sectional studies of ON patients in the acute stage (*n* = 1), after recovery (*n* = 3), and a few studies with a mixed group of ON patients with a wide range of time since onset (*n* = 3), including unknown time since disease onset (*n* = 1), as well as four longitudinal studies. The stimulus parameters utilized and results reported in these studies are summarized in Figure 4. Unless otherwise indicated in column *Stimulus: Type vs. Baseline; Fellow Eye Status*, the stimulus is generally presented in a block design where it is alternated with no visual stimulation (i.e., darkness) to both eyes as the baseline condition. Likewise, the fellow eye is patched or occluded with the use of light-proof goggles in all studies, unless specified in Figure 4. Some studies utilized red/green stimuli, as indicated in column *Stimulus: Color, Contrast and Flicker, Motion*, with red/green filter glasses for monocular stimulation. All ON studies scanned patients at 1.5 Tesla (Rombouts et al., 1998; Werring et al., 2000; Langkilde et al., 2002; Russ et al., 2002; Toosy et al., 2002; Levin et al., 2006; Jenkins et al., 2010; Mascioli et al., 2012) or 3 (Korsholm et al., 2007; Benoliel et al., 2017). The resolution of the functional scans are not clearly reported in the majority of the ON studies. In the reported studies, the in-plane resolution ranged from 0.9–3 mm with a slice thickness of 2–5 mm. Finally, visual areas seem to be delineated using retinotopy (Levin et al., 2006; Benoliel et al., 2017), anatomic landmarks (Langkilde et al., 2002; Korsholm et al., 2007), cytoarchitectonic maps (Korsholm et al., 2007), and/or a semi-automated contouring technique (Toosy et al., 2002), and data were analyzed in Talairach and Tournoux (Werring et al., 2000; Toosy et al., 2002; Levin et al., 2006; Mascioli et al., 2012; Benoliel et al., 2017) or Montreal Neurological Institute (Toosy et al., 2005; Korsholm et al., 2007; Jenkins et al., 2010) reference space.

fMRI studies of *acute* unilateral ON patients revealed that activity in their visual cortex is greatly reduced for monocular stimulation of the affected eye (Russ et al., 2002; Korsholm et al., 2007; Jenkins et al., 2010; Mascioli et al., 2012), and to a lesser extent the fellow eye (Toosy et al., 2005) compared to the control group. In addition to a reported 40% reduction in BOLD responses in the visual cortex from the affected eye (Russ et al., 2002), BOLD signals from LGN were also reduced (Korsholm et al., 2007), when compared to the fellow eye and to controls. Moreover, the affected eye in ON patients showed a greater deficit to a rapidly changing stimulus, meant to engage the magnocellular channel, than to slowly contrast reversing stimuli (Russ et al., 2002). This is most likely an indication that, in addition to parvocellular-dominated loss associated with central vision in early ON, both visual pathways may be affected in the setting of diverse patterns of visual field deficits.

Several studies further emphasize that abnormalities for the affected eye in the acute phase of the disease extend beyond the early visual cortex (V1/V2). The activity in the *ventral visual processing stream*, i.e., area LOC is consistently reported to be reduced at baseline (Korsholm et al., 2007; Raz et al., 2011). However, the results have been quite mixed regarding the BOLD responses in the *dorsal stream*, including the middle temporal visual area (MT/V5) in acute ON patients. Raz et al. (2011) reported that stimulating the affected eye with visual-motion stimuli revealed a deficit in MT from the onset of the disease, although the exact timepoint since onset during data collection is unknown. In contrast, another study found no difference in the pattern of BOLD activity within MT (but not so in V1) in response to stimulation of the affected eye in comparison to fellow eye (Mascioli et al., 2012). This apparent robustness of MT to acute visual deficits, or perhaps to low visual contrast, in the affected eye is intriguing, but the generality of this finding is still quite unclear. This study utilized sub-optimal stimuli (i.e., a central stationary checkerboard) and a small cohort (*n* = 6) with mixed causes of optic nerve damage. Finally, a separate study of acute ON reported that in response to flickering checkerboard stimulation the *fellow eye* produced abnormally *high* BOLD activation in dorsal regions of bilateral cuneus (Jenkins et al., 2010). In this case, the interpretation is suggestive of cortical compensatory mechanisms. Indeed, this effect remained significant even after adjusting for covariates that reflect afferent input such as optic nerve lesion length, VEP amplitudes and demographic characteristics (Jenkins et al., 2010). So, with regard to the dorsal visual cortex, the findings for acute ON remain unclear and become even harder to explain when considering the longitudinal deficits reported in these dorsal regions (considered below). Clearly, to resolve these contradictory findings, more studies need to focus on investigating the pattern of activation in extrastriate areas in response to stimuli these areas have a preferential bias for, such as stimuli with motion (i.e., for MT) and color, natural objects or scenes, and movies (i.e., for LOC).

Several longitudinal studies exist, and they suggest that the adaptive plasticity of the visual pathway as a whole is important for facilitating the spontaneous recovery of vision in ON patients. For example, fMRI measurement from the LGN and cortical visual areas (V1, V2, LOC) was reported at onset, as well as 3 months and 6 months from onset (Korsholm et al., 2007). The inter-eye difference in activation was shown to diminish (i.e., the fMRI signal of the affected eye increased) during recovery (Korsholm et al., 2007; Raz et al., 2011), with the affected eye showing normal visual acuity as early as 4 months from onset of the disease (Raz et al., 2011). However, since the VEP amplitude of the affected eye improved as the BOLD signal in LGN improved, the authors attributed this effect to the resolution of inflammation in the optic nerve rather than the emergence of compensatory activity within LGN or other visual areas (i.e., rewiring in areas receiving altered visual input). Similarly, the recovery of BOLD activity in early visual areas and LOC at 6 months is suggested to be a result of reduced inflammation of the optic nerve and the subsequent improvement in BOLD signal in LGN and VEP amplitude (Korsholm et al., 2007). Nevertheless, the mechanisms underlying VEP amplitude recovery despite progressive post-inflammatory degeneration and demyelination of the optic nerve is unclear. By contrast, other patterns of activity do suggest the existence of compensatory mechanisms within LGN to preserve visual function after insult to the optic nerve. The LGN of ON patients was seen to perform *above* normal upon stimulation of the fellow eye at baseline, but this disappears as BOLD activity in the affected eye normalizes, around 4–6 months from disease onset (Korsholm et al., 2007). In the same vein, Toosy et al. (2005) suggest a compensatory cortical activity to account for the improvement of *ventral stream* extrastriate regions (i.e., lateral temporal, fusiform) at 3 months from disease onset. The normalized BOLD measures correlated with clinical improvement, even after accounting for optic nerve structural deficits.

Despite the substantial recovery of vision in the months following disease onset, it is important to note that certain aspects of vision remain impaired. For example, the affected eye of ON patients 1 year after disease onset still shows ~20% reduction in BOLD amplitude in early visual areas (Langkilde et al., 2002; Russ et al., 2002; Levin et al., 2006; Benoliel et al., 2017). Also, delays in VEP latencies last for a prolonged, perhaps indefinite, period of time (Korsholm et al., 2007). In addition, a persistent deficit within MT (Raz et al., 2011), as well as weaker functional connectivity between early visual areas and MT/LOC are reported for the affected eye in comparison to fellow eye and controls (Benoliel et al., 2017). Although this needs to be further clarified, the findings outlined above imply that abnormally high activity in the *acute* stages of the disease (e.g., in MT or elsewhere in the dorsal stream) may actually predict a *lack* of recovery. Alternatively, the prolonged MT abnormalities could also reasonably be related to delayed VEPs, given the importance of temporal precision for visual motion processing.

Finally, the exact time points at which MRI and other brain measures improve for ON patients remains to be determined. For example, due to the lack of studies that investigate the activity of MT at systematic intervals from disease onset, it is difficult to determine the critical time points of disease progression within MT. More generally, it is still unclear exactly where, when, and if visual areas normalize, as some studies report it to take place within 1 month whereas others suggest 6 months. Moreover, the pattern of activation in extra-occipital regions, brain areas outside of the early visual areas, may be non-linear during the course of the disease. In other words, at one time point, an abnormally high activity in an extra-occipital region correlates with clinical deficit whereas at another time point it shows a BOLD deficit that is consistent with the reported clinical deficits. Therefore, further work needs to be conducted to understand the sequelae of BOLD alterations, and whether they are temporary (i.e., diminishes as visual function improves) or permanent (appears after the attack and remains during recovery and post-recovery phase) by regressing out the structural and functional optic nerve changes from the percent BOLD change we observe in visual areas.

### Correlation with specific ophthalmological measures

As was seen with glaucoma, the brain activity of ON patients often correlates with specific ophthalmologic indices of severity and allows us to better understand the factors influencing the changes in cortical activation. As such, in multiple studies, visual perimetry scores of the affected eye are highly correlated with fMRI responses from the early visual cortex (Russ et al., 2002; Korsholm et al., 2007; Jenkins et al., 2010), LGN (Korsholm et al., 2007), as well as LOC and peristriate cortex (Toosy et al., 2005) from the onset of disease. Correlations seen within LOC and peristriate cortex are regarded as true cortical alterations, and not evidence of separate defects, as they remained even after accounting for the structural deficit of the optic nerve (Toosy et al., 2005). BOLD activity from the visual cortex of recovered patients also corresponds to the residual deficit reported in perimetry scores (Langkilde et al., 2002). Finally, contrast sensitivity and Snellen visual acuity are also typically correlated with BOLD signal change in the visual cortex (Langkilde et al., 2002; Russ et al., 2002).

Moreover, optic nerve structural measures also correlate moderately well with fMRI responses in the affected eye of recovered ON patients, suggesting it is a good marker for chronic changes in the visual pathway of ON patients. A longitudinal study showed that BOLD activity in the visual cortex of acute ON patients at baseline corresponded with the optic nerve structural abnormalities (gadolinium-enhanced lesion length; Toosy et al., 2005). Surprisingly, at 3 months, reduced integrity of the optic nerve produced greater activity in extra-striate regions. However, by 12 months, residual abnormality in the optic nerve once again predicted a BOLD deficit in these extra-striate regions. Due to the constantly changing status of the optic nerve, at least in the early stages of ON, interpreting the biological meaning of the correlation of RNFL thinning/OCT measures with brain activity is difficult (i.e., are the discrepancies in the correlation pattern due to the lag of brain atrophy to RNFL thinning or robustness of visual cortex to alterations of the anterior visual pathway). Likewise, functional measures such as VEP latency and amplitude also show a low, but significant correlation with BOLD variables (Russ et al., 2002). Particularly, slow VEP latencies through the optic nerve in the affected eye, but not the fellow eye, of recovered unilateral ON patients were associated with decreased activation of the motion-related visual cortices such as V3 and MT (Benoliel et al., 2017).

### Visual stimuli

The importance of the choice of stimuli in revealing ON-related abnormalities in the occipital cortex and the related cerebral cortices is quite evident from the findings reviewed (Figure 4). In the acute phase of the disease, stimulating the affected eye resulted in a deficit in visual and extra-occipital regions with any of the following stimuli: checkerboard, static luminance-defined objects, or motion-defined objects (see Figure 4). Longitudinally, checkerboard or static luminance-defined objects subsequently showed recovery of BOLD signal from the early visual cortex, LGN, and LOC, as early as 1 month from disease onset. However, with the use of checkerboard and more complex motion-related stimuli, Raz and colleagues reported reduced activity within the visual cortex (especially V1) and MT, as well as abnormalities in connectivity between early visual areas and LOC to persist even after 12 months of disease onset. Hence, it is evident that the sensitivity of fMRI in detecting the deficits in optic neuropathies such as ON is partially limited by the stimuli and the fMRI paradigm chosen for the study.

Many of the early studies use simple photic stimulation (see Figure 4). This is not an ideal visual paradigm as often these studies report activity observed in widespread regions in the striate and extra-striate cortex and it becomes difficult to understand what the reported deficit and/or compensatory activity within these areas means in terms of visual function. The majority of the studies resorted to black and white checkerboards, particularly in the central visual field. However, ON can produce widespread and diffuse patterns of visual field loss, especially in the acute stages, and therefore utilizing standard full-field checkerboard stimuli may be more beneficial in identifying the deficits. It is also a reliable stimulus to activate the primary visual cortices but needs to be supplemented with other types of stimuli in order to study the activity of higher-order visual areas. Indeed, some studies used more complex stimuli to test aspects of central visual function such as object and motion perception. However, given that such stimuli were found only in 2 of the 12 articles we reviewed, results are hard to generalize. Moreover, no studies have used truly natural stimuli with objects and scenes. Although the reason is unclear, it may be these stimuli are relatively more difficult to use, or incompatible with dichoptic stimulation of the eyes. Of course, equally important is to consider the stimuli utilized during the baseline condition and the status of the fellow eye in dichoptic stimulation paradigms (see column *Stimulus: Type vs. Baseline; Fellow Eye Status* in Figure 4). Neither of these parameters are consistently controlled within the studies we reviewed. Emerging evidence suggests that the status of the untested eye can significantly impact the fMRI BOLD signal obtained due to potential inhibitory effects from that eye (e.g., Chadnova et al., 2018). Therefore, future studies should consider these issues more carefully.

## Traumatic Optic Neuropathy

TON is a rare but serious acute optic neuropathy resulting in partial or complete visual loss as a result of a lesion to the optic nerve due to trauma (i.e., a direct hit to the head or orbit in for example accidents or falls). It can be present unilaterally or bilaterally. TON can be classified as direct, via a penetrating injury, or more commonly indirect, when the force of the impact from head trauma is transferred to the optic nerve (Miliaras et al., 2013). The mechanical shearing of optic nerve axons and/or ischemia is thought to be the underlying mechanisms.

In TON, the affected eye(s) shows atrophy of the optic disc along with a severe reduction in visual acuity, visual field defects, and impaired color perception (Miliaras et al., 2013). Reduced RNFL and ganglion cell/inner plexiform layer thickness (Kyncl et al., 2019) are also seen at about 3–6 weeks following injury (Miliaras et al., 2013). These ophthalmological findings are generally normal for the fellow eye (Kyncl et al., 2019). However, pattern electroretinogram and pattern visual evoked potential, an electrophysiological measure of the function of ganglion cells and optic nerve, respectively, has been reported to be abnormal for both the affected and fellow eye for at least 3 years since onset. Despite the usefulness of these measures, there are limitations for use in TON. Due to the traumatic nature of the condition, TON patients are often unconscious or cognitively challenged in these assessments. Consequently, diagnosis and intervention for early loss can be delayed for days.

As a result, recent studies are employing MRI to study TON as it allows us to directly investigate the impact on the optic nerve irrespective of the cognitive status of the patient. There has been some progress in applying modern structural MRI techniques (Takehara et al., 1994; Kyncl et al., 2019), including diffusion tensor imaging to characterize the optic nerve in closed head trauma (Yang et al., 2011; Bodanapally et al., 2013, 2015; Li et al., 2014). However, the results are too sparse to allow meaningful generalization. Also, the effect of the structural alternations observed in the optic nerve on the activity of subsequent visual brain activity needs to be studied.

### Visually driven functional brain measures

Unlike glaucoma and ON, TON has barely been studied using visually-driven fMRI. However, visually-driven fMRI has been utilized in a single case report of an individual with an optic nerve lesion due to an injury to the orbit. fMRI scans utilizing a checkerboard stimulus performed on the fellow eye of an individual ~9–10 months since TON onset revealed a reduction in the activity of the visual cortex. Cortical activity is reported to gradually increase but does not recover completely as deficits persist even 4 years after onset (Kyncl et al., 2019). It is important to note that the affected eye was not stimulated due to the extent of damage and inability to maintain fixation on the chosen stimulus. Finally, there are a few studies that have employed functional MRI to study a clinical manifestation similar to TON called open globe injury, but at rest. Cortical brain activity in acute open globe injury patients studied with various resting-state fMRI techniques revealed functional network dysfunction in the bilateral visual cortex/calcarine/lingual/cuneus, temporal and dorsal visual pathway (Tan et al., 2016; Ye et al., 2018), as well as in numerous higher-level brain regions such as supplementary motor area (Wang et al., 2017), frontal area and cerebellum (Huang et al., 2016). Currently, it is impossible to distinguish whether the resulting brain activity is a result of poor visual input or compensatory activity due to the reorganization of cortical networks.

Although it remains to be shown that visually-driven fMRI is a useful methodology for studying TON patients, preliminary fMRI results suggest that it is possible to employ this technique to study unilateral and bilateral TON over time. Moreover, measuring spontaneous BOLD signals during the resting state may be especially valuable in this context. The minimal demands placed on patients during resting-state scans make this technique very feasible. Nevertheless, the lack of visually-driven TON studies can also be attributed to the rarity of the condition. It is difficult to recruit a sufficient number of patients and to ensure that the patients are at a similar stage of disease progression as TON is a rapidly changing condition. Moreover, the lack of consciousness in TON patients can make it difficult to instruct patients about the task they may be expected to perform while in the scanner. It is necessary to design visual stimuli carefully and test the MRI protocol extensively before use in TON patients to ensure smooth functioning without patient input. Finally, MRI is an expensive tool with reduced accessibility. Therefore, unless a well-thought-out system is in place, diagnosing and assessing the severity of a rapidly changing optic neuropathy like TON may be difficult.

Despite the challenges, TON results in severe consequences, and an improved understanding of the functional alterations in the visual pathway over time is needed since there is no treatment for TON at this time. Furthermore, correlating the functional brain responses to clinical measures longitudinally may provide prognostic insights. Importantly, this could be useful in identifying the factors that predict recovery of vision in some TON patients and support any future developments in drug treatment options. Clinicians could then better choose a treatment plan (e.g., natural recovery or intervention).

## Discussion

Despite some inconsistencies and the low sample size of some of the studies reviewed here, visually-driven fMRI has been shown to be sensitive and reliable in identifying functional loss of brain activity as a result of glaucoma- or ON-specific visual impairment(s). fMRI also appears promising for studying TON-induced deficits. The visually-driven fMRI literature for glaucoma patients consistently reported reduced activity in the bilateral occipital lobe, especially for peripheral visual field stimulation. In association with that, there is evidence that the magnocellular layers of the LGN are affected early in glaucoma patients, and this seems to be the only parameter that successfully differentiates glaucoma patients in the early stages from healthy normal controls. BOLD signal within V1 in response to central field stimulation is heavily dependent on the severity of glaucoma. The effect of glaucoma on higher visual areas at onset and during the progression of the disease is still unclear.

In comparison, for ON patients, reduced BOLD activity has been reported in the LGN, visual cortex, LOC, and MT, although a study reports MT to be hyperactive in the acute phase. Also, the abnormalities are more pronounced in the acute stage of the disease. Brain activity, especially within LGN, visual cortex and LOC, is reported to increase and then normalize within 3–4 months of disease onset, and this is suspected to underly the spontaneous recovery in clinical visual function in ON patients, but more direct evidence is needed. It is notable that motion perception in particular appears to remain impaired in ON patients.

It appears that RNFL measures correlate better with brain activity in early glaucoma patients. This may be due to the high reproducibility of optic nerve measurements and the marked/rapid changes the retinal nerve fiber layer undergoes at the early stages of glaucoma. Whereas, perimetry seems better for ON, likely because of the better sensitivity of perimetry to central vs. peripheral vision.

Similar to glaucoma and ON, visually-driven fMRI results from a single TON patient suggest reduced activity within the visual cortex at disease onset, followed by minimal recovery of signal. Future studies of TON patients should take inspiration from the visually-driven fMRI work conducted with glaucoma and ON patients as they have shown that optic neuropathy- induced alterations can be seen in subcortical and cortical visual areas, including retinotopic-specific alterations where applicable, from the onset of the disease.

Compared to glaucoma, ON seems to have a stronger basis for functional cortical plasticity, however, it has not been extensively explored in either of the patient groups. This is yet to be studied in TON patients. Future studies should investigate the physiological basis for compensatory-like BOLD responses in higher-level visual areas, as reported for ON patients. The cortical circuitry underlying such activity and its role in resolving vision are unknown. We will also need to investigate whether the deafferented cortical regions are rewired to process information from intact regions of the retina (i.e., enlarged receptive fields), or whether the input of the fellow eye is strengthened in these regions to make up for the lack of input from the affected eye, or is it some combination of both. The documented “remapping” of parafoveal regions of V1 in POAG provides a precedent for these effects, at least in the context of slow progressive vision loss.

Moreover, there are still many other questions that need to be investigated in glaucoma, ON, and TON patients. For all conditions, future progress would be enhanced by comparison of multiple stimuli that maximally drive various subcortical channels, visual brain areas, and cortical pathways. In addition to the known association between central/peripheral vision and parvocellular/magnocellular input, it will be interesting to use more complex stimuli that are simply more effective at driving higher-tier areas. Testing directly for dysfunction of higher- order visual functions such as object recognition has already been achieved by a few studies of ON. Further, using the same set of stimuli to study multiple patient groups would allow direct comparison of activation patterns. In addition, to increase the feasibility of acquiring data from the affected and the fellow eye of patients with central vision loss like ON and TON, the stimuli utilized in these studies should be designed with care. Utilizing full-field stimuli, larger fixation point and/or long crosshairs, as well as eye-tracking could aid ON and TON patients maintain fixation with the affected eye for the duration of the scan. The potential role of fMRI for POAG is quite different than ON and TON for several reasons. The slow onset of peripheral field loss poses a serious challenge for early detection, and fMRI does not seem likely to provide an easy solution to that issue. However, once patients are diagnosed with POAG it is conceivable that fMRI could be optimized for sensitivity by focusing on the magnocellular pathway, and relying on hardware to stimulate the peripheral field as much as possible. Nonetheless, the cortical magnification of the central field is a very important feature of fMRI data, suggesting that greater clinical impact is likely for conditions like ON and TON. This “enrichment” of sensitivity to central vision loss is a big advantage, as is the feasibility of scanning before, during and after recovery. Finally, for all conditions, since brain areas do not work in isolation, studying the functional connectivity in visually-driven activation patterns of the brain, in addition to BOLD patterns and strength, is crucial.

In conclusion, to obtain a more complete understanding of the optic neuropathies, it is important to continue investigating them using neuroimaging techniques that allow us to study the effect of the disease on the entire visual pathway. The potential remains for detecting early or subtle changes, possibly before structural or functional eye measures. Likewise, it may help identify reliable neuroimaging parameters that can serve as prognostic measures for visual function recovery rate in ON and TON patients. This may help promptly choose appropriate participants for additional drug therapy. Finally, it may allow us to identify neuroimaging biomarkers that differentiate acute optic neuropathies from chronic optic neuropathies. Hopefully other studies utilize visually-driven fMRI to investigate glaucoma, ON and TON in a novel way to obtain valuable information about these blinding diseases.

## Author Contributions

JM and AS conceived the idea. JM, AS, and SS developed the idea. SS wrote the manuscript in consultation with JM. All authors provided feedback and helped shape the manuscript. All authors contributed to the article and approved the submitted version.

## Funding

This project was supported by funds from U.S. Department of Defense CDMRP Vision Research Program Grant # W81XWH1910853.

## Abbreviations

BOLD: Blood oxygenation level-dependent signal
fMRI: functional magnetic resonance imaging
LGN: Lateral geniculate nucleus
LOC: Lateral occipital cortex
MT/V5: Middle temporal visual area
ON: optic neuritis
OCT: Optical coherence tomography
POAG: Primary open-angle glaucoma
RAPD: Relative afferent pupillary defect
RNFL: Retinal nerve fiber layer
TON: traumatic optic neuropathy
VEP: Visually evoked potentials
V1: Primary visual cortex
V2: Secondary visual cortex
V3: Tertiary visual cortex.
