# Supplementary material for: Visually driven functional MRI techniques for characterization of optic neuropathy
Source: Front Hum Neurosci. 2022 Sep 9;16:943603. doi: 10.3389/fnhum.2022.943603 (PMC9500431; doi:10.3389/fnhum.2022.943603)
Supplement: Supplementary file 1 [file Table_1.DOCX]

Supplemental Table 1. Patient demographic and disease information from ON studies reviewed in this paper. Unless stated, the studies have excluded MS and bilateral ON patients. Studies that utilized acute ON patients are at the top, followed by studies of recovered ON patients; within which, it is sorted according to time since onset.

MS = multiple sclerosis

| **Author, Year** | **Number of ON Patients** | **Age (Range/Mean (± SD)** | **Stage & Type of ON; # of Occurrences** | **Mean Time from Onset at data collection** |
| --- | --- | --- | --- | --- |
| 1. Jenkins   et al., 2010 | 28 | 32 | Acute, typical clinically isolated *unilateral* ON | Same-day or within 1 month |
| 1. Russ   et al., 2002 | 20 | 18 - 46  (mean: 28.8 ± 6.4) | Acute, progressive *unilateral* ON | **Baseline:** Within 3 days  **Subsequently:** Unclear (~1- 2 months) |
| 1. Korsholm   et al., 2007 | 19 | 18 - 45  (median: 31.5) | Acute, clinically isolated ON  (Includes relapsing -emitting MS with a normal fellow eye) | **Baseline:** 9 - 42 days (median: 19 days)  **Subsequently:** 2 weeks, 3 and 6 months after baseline |
| 1. Toosy   et al., 2005 | 21 | 33.8 | Acute, typical *unilateral* ON; one episode | **Baseline:** 3 week  **Subsequently:** 2 weeks, 1, 2, 3, 6 and 12 months after baseline |
| 1. Raz   et al., 2011 | 13 | 18 – 41  (mean: 28.9 ± 6.6) | *Unilateral* ON; one episode | **Baseline:** Unclear (~ <1 month)  **Subsequently:** Followed for up to 1 year |
| 1. Mascioli   et al., 2012 | 8 | 46 | ON  (Includes MS, optic nerve head damage connected to maxillofacial injury, ischemic ON, and idiopathic) | Unknown |
| 1. Rombouts   et al., 1998 | 9 | 23 - 43  (mean: 33) | Acute & Recovered, *unilateral* ON  (Includes relapsing-remitting MS) | 7 days - 12 years |
| 1. Levin   et al., 2006 | 8 | 19 - 53  (median: 30) | Acute & Recovered, *unilateral* ON; acute episode | 10 days - 5 years |
| 1. Werring   et al., 2000 | 7 | 37.8 | Recovered, typical *unilateral* ON; one episode | 6 months - 14 years |
| 1. Benoliel   et al., 2017 | 11 | 18 - 45  (mean: 27.84 ± 7.36) | Recovered, ON | 1 - 2 years (mean: 13.6 months) |
| 1. Toosy   et al., 2002 | 8 | 37.1 | Recovered, *unilateral* ON, one episode | 1 - 14 years (mean: 7.2 months) |
| 1. Langkilde   et al. 2002 | 9 | 35 - 64  (median: 48) | Recovered, ON; one or more episodes  (Includes clinically definitive or possible MS and bilateral ON) | 5 - 7 years |
